# How far can the self be extended? Automatic attention capture is triggered not only by the self-face

**DOI:** 10.3389/fpsyg.2023.1279653

**Published:** 2023-11-03

**Authors:** Anna Żochowska, Michał J. Wójcik, Anna Nowicka

**Affiliations:** ^1^Laboratory of Language Neurobiology, Nencki Institute of Experimental Biology, Polish Academy of Sciences, Warsaw, Poland; ^2^Department of Experimental Psychology, University of Oxford, Oxford, United Kingdom

**Keywords:** self-prioritization, attention, familiarity, saliency, ERP

## Abstract

The preferential processing of self-related information is thought to be driven by its high level of familiarity. However, some behavioral studies have shown that people may exhibit a preference for initially unfamiliar stimuli that have been associated with themselves arbitrarily. One of the key questions that needs to be addressed concerns the role of early attention in the prioritization of newly acquired information associated with the self. Another question is whether both highly familiar as well as new information referring to a subjectively significant person (i.e. close-other) benefits from preferential attentional processing. We aimed to tackle both questions by investigating the neural mechanisms involved in processing extremely familiar stimuli, like one’s own face or the face of a close-other, as well as stimuli (abstract shapes) that were newly linked to each person. We used a dot-probe paradigm that allowed us to investigate the early stages of attentional prioritization. Our analysis of the N2pc component unveiled that attention was automatically captured by the self-face, a shape associated with oneself, and the face of the close person. However, a shape associated with the close-other did not elicit the same attentional response, as the N2pc was absent. Thus, both the self-face and information referring to the extended self (self-assigned shape, close-other’s face) benefit from preferential early and automatic attentional processing.

## 1. Introduction

In the complex social world humans live in, they must be able to select only a part of incoming information for further processing. Numerous studies have demonstrated the preferential processing of self-relevant stimuli at various stages of the processing hierarchy (e.g., Cunningham et al., 2008; Keyes and Brady, 2010; Tacikowski and Nowicka, 2010; Devue and Brédart, 2008; Sui et al., 2012, 2014; Kotlewska and Nowicka, 2015, 2016; Schäfer et al., 2016; Macrae et al., 2018; Żochowska et al., 2021). These stimuli, including one’s own face, personal possessions, and so forth, serve as means through which individuals differentiate themselves from others (James, 1890; Belk, 1988; Dittmar, 1992). A fundamental assumption of recent theoretical perspectives on self-prioritization is that stimuli related to oneself actively exert an impact on the processing of information, encompassing initial attentional functions (Humphreys and Sui, 2016; Sui and Rotshtein, 2019).

Consistent with the latter concept, recent studies have demonstrated the early and automatic capture of attention by an individual’s own face (Wójcik et al., 2018, 2019; Bola et al., 2021). In these studies, images of faces – including one’s own face – were presented as irrelevant stimuli within a dot-probe task. This task facilitates the investigation of the attention-grabbing characteristics of distractors that should be disregarded, as measured by the N2 posterior-contralateral (N2pc) component of event-related potentials – ERPs (Luck and Kappenman, 2012). Analysis of this electrophysiological marker of covert attentional shifts (Eimer and Kiss, 2008; Sawaki and Luck, 2010) revealed that the self-face attracts attention automatically and involuntarily (Wójcik et al., 2018, 2019; Bola et al., 2021). This was also the case for the self-face made invisible through backward masking (Wójcik et al., 2019; Bola et al., 2021).

The rapid and involuntary capture of our attention by our own face could be attributed to the extreme familiarity associated with it. This familiarity stems from the frequent exposure to our reflection in mirrors and on photographs (Bortolon et al., 2018). If indeed familiarity plays a pivotal role in directing our attention, then we should also anticipate a similar shift in attention toward other well-known faces. However, the degree of familiarity related to faces can be seen as a spectrum, spanning from mere visual familiarity (e.g., the face of a new neighbor or colleague) to extreme familiarity (e.g., our own face or that of our mother), with graded/varying levels of familiarity in between (e.g., the faces of famous actors or individuals we personally know). Additionally, numerous factors influencing the overall level of familiarity may interact with each other. These factors include the frequency of exposure to a particular face, the presence and intensity of emotional bonds, and the extent of personal information available about the individual whose face we are encountering (Ramon and Gobbini, 2018).

One of the previous studies utilizing dot-probe ERP methodologies focused on the role of mere visual familiarity of faces (Bola et al., 2021). This type of familiarity was established by initially exposing participants to unfamiliar faces before the experiment, followed by recurrent displays of the same face throughout the course of the experiment (Bola et al., 2021). A comparison was made between the processing of these visually familiar faces and the processing of the participants’ own faces. Attention was automatically and involuntarily captured by participants’ own faces as evidenced by the N2pc. In contrast, this ERP response was notably lacking in the case of faces that acquired visual familiarity through repeated presentations (i.e., faces that gained increased intra-experimental familiarity). This finding undermines the role of mere visual familiarity as a factor driving attentional prioritization.

Hence, a question arises whether a pre-experimentally highly familiar non-self-face (such as the face of a close-other) can benefit from preferential attentional processing in the early stages. Beyond its exceptional familiarity, a close-other’s face also shares other attributes with the self-face. For instance, gazing upon such a face might evoke emotions or activate semantic knowledge about the close-other individual (Ramon and Gobbini, 2018). Investigating attention capture for both types of faces (self and close-other’s) within the dot-probe task would further address the question of whether familiarity indeed plays a role in the observed attentional prioritization or if other factors are at play—like the activation of self-representation (Devue and Brédart, 2008) or the emotional aspects linked with the processing of familiar faces (Gobbini et al., 2004; Gobbini and Haxby, 2007).

A compelling approach to examining the role of familiarity in influencing early attention capture can be found in studies that employ perceptual matching tasks to associate novel information with both oneself and others (Sui et al., 2012, 2013, 2014). Within these investigations, basic geometric shapes were arbitrarily linked to the self, a friend, and an unfamiliar other. Subsequently, participants were tasked with determining whether shape-label pairings aligned with the assigned associations. The immediate self-prioritization effect emerged for shapes associated with oneself, demonstrated by notably quicker responses (i.e., shorter reaction times) for matches involving the self-associated shape and label compared to other shape-label pairings.

The advantage of processing information newly attributed to one’s own identity has been consistently validated across a diverse range of studies through behavioral markers such as reaction times (RTs), accuracy, and sensitivity scores (Sui et al., 2012, 2013, 2014; Frings and Wentura, 2014; Mattan et al., 2015; Schäfer et al., 2015, 2016; Macrae et al., 2017; Yin et al., 2019; Orellana-Corrales et al., 2021). Nevertheless, overt behavioral measures reflect the ultimate results of a series of processes, starting with sensory stimulation, followed by decision-making processes, and culminating in a motor response. In contrast, temporally sensitive ERPs provide direct insights into the initial stages of information processing.

In a ERP study that utilized the perceptual shape-label matching task, increased early (N1) and late (P3) components were observed in the self-condition in comparison to conditions involving friends and strangers (Sui et al., 2023). It is worth noting that this study required participants to concurrently process the self-associated arbitrary stimuli and familiar verbal labels with established meanings. Consequently, these labels could potentially trigger the activation of self-representation, which, in turn, steers attention toward newly acquired self-relevant stimuli. Hence, the swift integration of shape and label during the decision-making process might consequently give rise to the self-prioritization effect (Sui et al., 2023). In line with the later concept, in the absence of familiar labels, a lack of self-prioritization was found for abstract shapes randomly assigned to the self (Żochowska et al., 2023). More specifically, there was no discernible difference in P3 amplitudes between the self-assigned shape and the shape assigned to a close-other. Given that the P3 is conventionally considered a neural indicator of attention allocation (Polich, 2007), this finding implies a comparable allocation of attentional resources (i.e., analogous top-down attention). However, an essential question arises whether new information that is arbitrarily assigned to the self vs. a close-other, presented without any familiar label, will automatically capture and bias early (i.e., bottom-up) attention (as revealed by the N2pc).

Moreover, there is an ongoing debate about necessary conditions that lead to the biasing of subsequent processing by newly acquired self-assigned information. Some evidence suggests that self-relevance enhances the processing of stimuli only when the participant’s task directly pertains to previously established shape-label associations (Caughey et al., 2021). For instance, self-assigned shapes were categorized faster than shapes associated with a friend when participants needed to identify the presented stimulus (self or friend), but this effect was not observed when they were tasked with judging the position of shapes on the screen (above or below fixation). An analogous pattern of results emerged for arbitrary objects assigned to the self and a friend (Falbén et al., 2019). Similarly, the self-prioritization effect did not emerge when participants were merely required to detect the self-associations (Desebrock et al., 2022), or when the connections of the stimuli with the self were generally unrelated to the ongoing task (Woźniak and Knoblich, 2022). This line of research highlights the conditional automaticity in the processing of newly acquired self-associated information and implies a predominant top-down guidance of attentional processes. However, in the case of self-face, automatic attention capture was not contingent on whether the self-referential information was task-relevant as faces were to-be-ignored stimuli (Wójcik et al., 2018, 2019; Bola et al., 2021). In light of the mentioned findings, investigating newly learned self-assigned stimuli presented as task-irrelevant elements in a dot-probe paradigm could provide more arguments in favor of or against the role of task-relevance in self-prioritization.

The goals of the present study were twofold. First, our aim was to investigate the automatic attention capture elicited by both the self-face and the face of a close-other. This approach allows us to determine whether an emotionally salient and extremely well-known non-self-face is processed at the early stages in a similar manner as the self-face. Second, we explored whether an abstract shape can trigger involuntary attentional capture following a brief association with the self. Additionally, we examined whether the same effect is observable when the shape is linked to a close-other. This enabled us to test for the plausible self-specificity of early attentional capture.

## 2. Materials and methods

### 2.1. Participants

The study included 35 participants (19 females, 16 males), whose ages ranged from 21 to 34 years (*M* = 28.3; *SD* = 3.2). The G*Power 3 software (Faul et al., 2007) was employed to determine the necessary sample size on the basis of the data acquired in our previous dot-probe N2pc studies with the self-face (Bola et al., 2021). Cohen’s d was estimated using paired-samples two-tailed t-tests on the mean amplitudes of contralateral and ipsilateral waveforms and their standard deviation [with α = 0.05, power (1-β) = 0.80]. It was equal to −0.67. Thus, a sample size of 20 participants would be necessary to detect the N2pc. However, the group size was enlarged to 35 in order to ensure sufficient sample size in the case of the risk of data loss or exclusion (on the basis of high number of artifact-contaminated EEG trials).

Handedness assessment according to the Edinburgh Handedness Inventory (Oldfield, 1971) revealed that 33 participants were right-handed. All participants had normal or corrected-to-normal vision and had no history of mental or neurological disorders. In order to maintain consistency in visual stimuli, photographs of participants and their selected close-others were devoid of glasses or distinct facial marks, ensuring alignment with images from the Chicago Face Database – CFD (Ma et al., 2015).

### 2.2. Stimuli

The stimuli employed in the experiment encompassed pairs of face photographs or abstract shapes presented bilaterally. These stimuli sets were individually tailored for each participant. Participants freely selected their close-others based on subjective closeness and significance. However, to ensure control over gender-related effects, the gender of close-other faces has to be matched with that of the participant. This selection approach has been consistent with earlier studies (e.g., Tacikowski et al., 2011, 2013; Cygan et al., 2014; Kotlewska and Nowicka, 2015, 2016; Kotlewska et al., 2017; Nijhof et al., 2018; Cygan et al., 2022; Żochowska et al., 2022, 2023; Amodeo et al., 2023). Among the participants, 23 individuals opted for a friend, 9 for their sibling, and 3 for their partner as their chosen close-other. Prior to the study, photographs were taken of each participant’s face and the face of their selected close-other, both exhibiting neutral expressions. Additionally, 11 photographs of unknown neutral faces were sourced from the CFD (Ma et al., 2015). The editing procedure was standardized for all photos, involving grayscale conversion, background removal, and cropping to encompass only facial features (the face oval excluding hair and ears). Subsequently, the photos were resized to occupy a visual angle of 6.31 × 8.38 and adjusted for mean luminance using Adobe Photoshop^®^ CS5 (Adobe, San Jose, CA).

In addition to face photos, participants were presented with abstract shapes. Unlike previous studies that used few simple geometric shapes, this study required a larger number of shapes (i.e., 13, matching the number of faces). These abstract shapes were carefully generated to match the area of the face oval and the luminance of the faces. This matching was crucial to control low-level visual features across all stimuli used in the study. Pseudo-random assignment of shapes to self, close-other, and unknown conditions was carried out at the group level. Depending on the block and trial type, pairs of stimuli were configured to display self-face/shape and unknown face/shape, close-other’s face/shape and unknown face/shape, or two unknown faces/shapes. The positioning of the inner edge of faces/shapes on the screen was at a distance of 3° from the fixation cross on both sides. This spacing allowed for effective detection of horizontal eye movements and the reduction of trials contaminated by such artifacts, following the practices of previous studies (Meyberg et al., 2017; Wójcik et al., 2018, 2019; Bola et al., 2021). This approach was reinforced by recent ERP research indicating the relative constancy of the N2pc across different eccentricities, even with stimuli placed at the midline (Papaioannou and Luck, 2020).

### 2.3. Procedure

Seated comfortably in a softly lit and sound-insulated room, participants were positioned 57 cm away from the computer monitor (DELL Alienware AW2521HFL, Round Rock, Texas, United States). Following this, as the EEG electrode cap was being placed and electrode impedances were adjusted. During the impedance adjustment, participants were shown on the monitor two distinct shapes, assigned to the participant and their chosen close-other, and were asked to learn these assignments. The average duration of the assignment learning was 24 min (*SD* = 6 min). Just prior to commencing the dot-probe task, participants were asked to sketch the shapes assigned to themselves and their close-other, a step taken to verify the efficacy of the learning process.

Upon reading and confirming their understanding of the instructions displayed on the screen, participants initiated the experiment by pressing a response button. Throughout the task, a chinrest ensured consistent head positioning and viewing distance.

**Figure 1:**
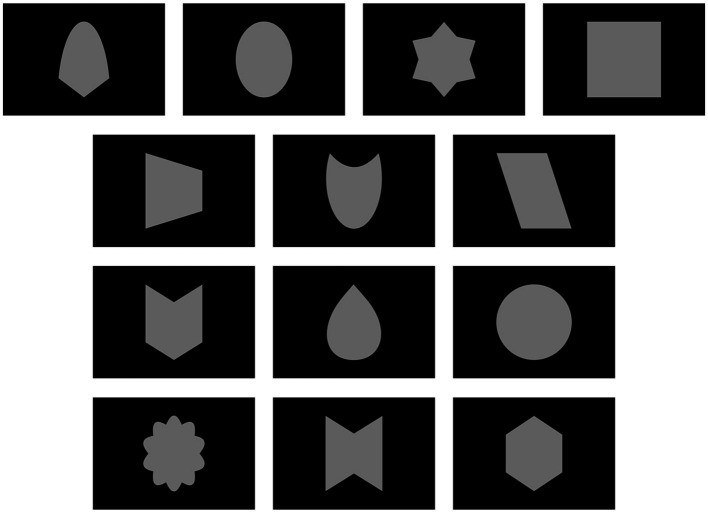
The shapes employed in this study.

Stimuli presentation was facilitated by Presentation software (Version 18.2, Neurobehavioral Systems, Albany, CA). Each experimental session comprised four blocks, with each block featuring pairs of either faces or shapes. There were two blocks with self-related stimuli and two blocks with close-other related stimuli. In the self-face/shape and close-other’s face/shape blocks of the dot-probe task, the self-face/shape or close-other’s face/shape was consistently paired with one of the 11 unknown faces/shapes. The unknown face/shape was presented contralateral to the self- or close-other’s face/shape. In each block, those pairs were intermixed with pairs of unknown faces/shapes. The order of these blocks was pseudo-randomized at the group level, ensuring equal likelihood for each block to occupy positions 1 through 4 (Figure 1).

**Figure 2:**
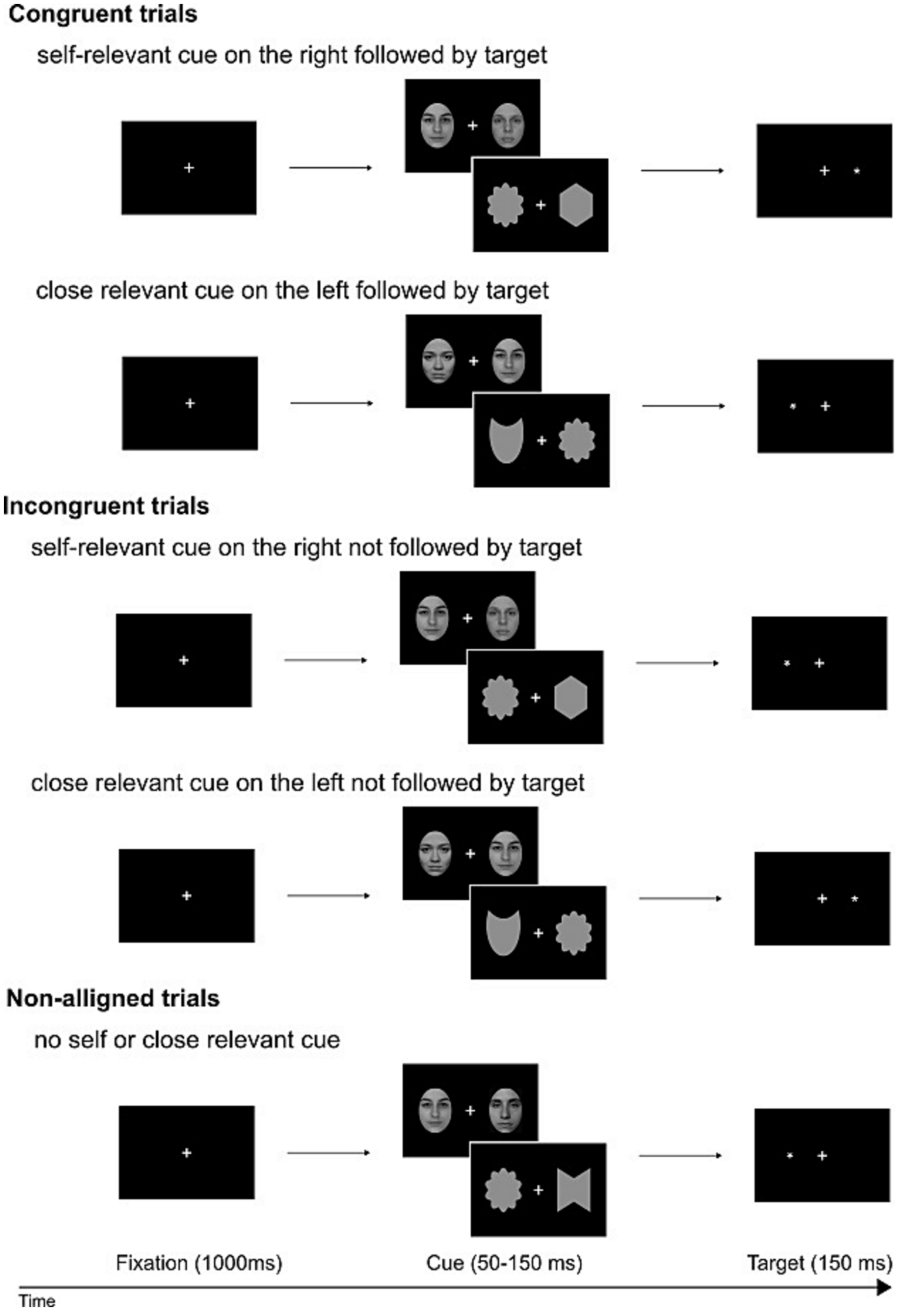
Schematic representation of dot-probe trials: congruent, incongruent, and non-aligned. Pairs of faces/shapes were interspersed and presented in a pseudo-random manner. These pairs, both for faces and shapes, fell into three categories: self and unknown, close-other and unknown, and two unknowns. Potentially salient faces/shapes (self- or close-other related) and unknown faces/shapes were presented to the right or to the left of fixation cross. Participants were instructed to disregard the faces/shapes and instead indicate the location of a subsequently displayed dot, reporting whether the target dot appeared on the left or right side with a manual response. The provided example photos of faces feature one of the co-authors and two members of our lab (who willingly consented to the use of their images).

The initiation of each experimental trial was marked by the appearance of a fixation cross (measuring 0.4 × 0.4 of visual angle), maintained throughout the trial. After 1,000 ms, a bilateral display of face/shape pairs appeared against a black backdrop. These pairs remained visible for 50–150 ms (average of 75 ms). Following this, the probe, represented by an asterisk (measuring 0.3 × 0.3 of visual angle), emerged in the visual field previously occupied by the self-face/shape or a close-other’s face/shape (in congruent conditions). In incongruent conditions, the probe appeared in the opposite visual field, while in non-aligned conditions, it took the place of one of two unknown faces/shapes presented bilaterally. The sequence of trials and the assignment of stimuli (face, shape) and conditions (self, close-other) were all pseudo-randomized. Each experimental condition comprised 132 trials. A graphical representation of the sequence of events in a single trial is shown in Figure 2.

Participants were instructed to promptly and accurately indicate the side of the probe’s appearance by pressing the corresponding button with their left or right index finger. They were also directed to maintain fixation on the cross and disregard the cues (faces/shapes). In each block, half of the trials with salient cues were congruent, and half were incongruent, with the order randomized. At the conclusion of the experimental session, the photographs of participants’ faces and those of their close-others were deleted from the computer’s storage.

### 2.4. EEG and EOG recordings

Continuous recording of the electroencephalogram (EEG) was conducted utilizing a set of 64 Ag-AgCl electrodes equipped with electrical shielding. These electrodes were affixed to an elastic cap (ActiCAP, Munich, Germany) and positioned in accordance with the extended 10–20 system (Jasper, 1958). To identify ocular artifacts, vertical and horizontal electrooculograms (EOGs) were registered through bipolar electrodes placed both above and below the right eye and at the external canthi of both eyes. Electrode impedances for the EEG were maintained below 10 kΩ. Amplification of the data was accomplished via a BrainAmp MR plus amplifier (Brain Products, Munich, Germany), with subsequent digitization using BrainVisionRecorder^®^ software (Brain Products, Munich, Germany) at a sampling rate of 500 Hz. The EEG signal was recorded with reference to an average computed from all channels (a calculation was done by the amplifier hardware).

### 2.5. Analyses of behavioral data

Behavioral data analysis focused on reaction times (RTs) for manual responses to target dots, reflecting attention shifts in the dot-probe task. This analysis was conducted using the JASP software package (Wagenmakers et al., 2018). Inclusion criteria involved trials with accurate responses taking place between 100 ms and 1,000 ms post dot presentation. Percentage of outliers was as follows, for congruent and incongruent trails, respectively – self-face: 2.6 and 2.6%, self-assigned shape: 1.9 and 3.5%, close-other’s face: 2.1 and 3.3%, shape assigned to a close-other: 2.8 and 2.9%. Given the right-skewed distribution of RTs, median RTs were computed for each participant and experimental condition, while response accuracy was calculated as a percentage of correct responses.

The analysis of behavioral data centered on the factor of congruency, defined by the side of dot presentation relative to the self/close-other’s face/shape (congruent vs. incongruent trials). Of particular interest was the impact of congruency and cue type, i.e., whether the target dot (probe) followed a self- or close-other-related face/shape. Thus, median RTs for congruent and incongruent trials were directly compared through paired-samples t-tests, performed separately for each experimental condition (self-face, close-other’s face, shape assigned to self, shape assigned to close-other). Reporting of results encompassed median RTs and mean accuracy values, accompanied by their standard deviations (as *Md ± SD* and *M ± SD*, respectively).

The conventional approach of null-hypothesis significance testing was supplemented by the incorporation of Bayesian analysis techniques, in which Bayes factors (BFs) were computed using the JASP software (Wagenmakers et al., 2018). It is important to highlight that BF_10_ assesses the degree of support for both the alternative and null hypotheses based on the available data. For all Bayesian tests, a medium prior scale (Cauchy scale 0.707) was employed. In brief, a *BF*_10_ falling between 1 and 3 suggests anecdotal/weak evidence in favor of the alternative hypothesis (H1), while values between 3 and 10 indicate moderate evidence, between 10 and 30 indicate strong evidence, between 30 and 100 indicate very strong evidence, and values exceeding 100 suggest extreme evidence (Lee and Wagenmakers, 2014). Conversely, with respect to low *BF*_10_ values, a range of 0.33 to 1 indicates anecdotal/weak evidence in favor of the null hypothesis (H0), 0.1 to 0.33 suggests moderate evidence, and 0.03 to 0.1 implies strong evidence for the absence of an effect. Finally, a *BF*_10_ between 0.01 and 0.03, as well as values lower than 0.01, provide very strong and extreme evidence for the absence of an effect, respectively.

### 2.6. ERP analysis

The EEG data underwent offline analysis using BrainVision Analyzer^®^ software (Version 2.2, Brain Products, Gilching, Germany). EEG data from 62 channels was re-referenced offline to the algebraic average of signals recorded at the left and right earlobes. The data was notch filtered at 50 Hz and band-pass filtered within the range of 0.01 to 30 Hz using a 2nd order Butterworth filter. Following this, the EEG signal was segmented into epochs of 600 ms, spanning from 200 ms before to 400 ms after the onset of cues. The duration of these segments was constrained due to the immediate presentation of dots that followed significant cues and led to participants’ motor responses. Baseline correction against the mean voltage during the 200 ms pre-stimulus interval was applied to these epochs.

We excluded from analysis epochs containing artifacts like vertical eye movements and blinks (defined by a voltage change exceeding 75 μV within a 200 ms interval in the VEOG channel), horizontal eye movements (indicated by a voltage change surpassing 75 μV within any 200 ms interval in the HEOG channel), and other aberrations (e.g., voltage steps exceeding 50 μV, voltage changes exceeding 100 μV within any 200 ms interval, amplitudes greater than 200 μV or lower than −200 μV, and activity within 100 ms intervals falling below 0.5 μV).

Due to the substantial presence of artifacts, four participants were omitted from the analysis, as more than 50% of trials were rejected. Consequently, the ERP analysis was conducted on a reduced sample of 31 participants. Within this group, approximately 12.78% of trials per participant were rejected on average. Following the outlined preprocessing procedures, the data was partitioned based on the conditions (self, close-other), the nature of stimuli (face, shape), and the side of presentation of self/close-other’s face/shape (left, right).

**Figure 3:**
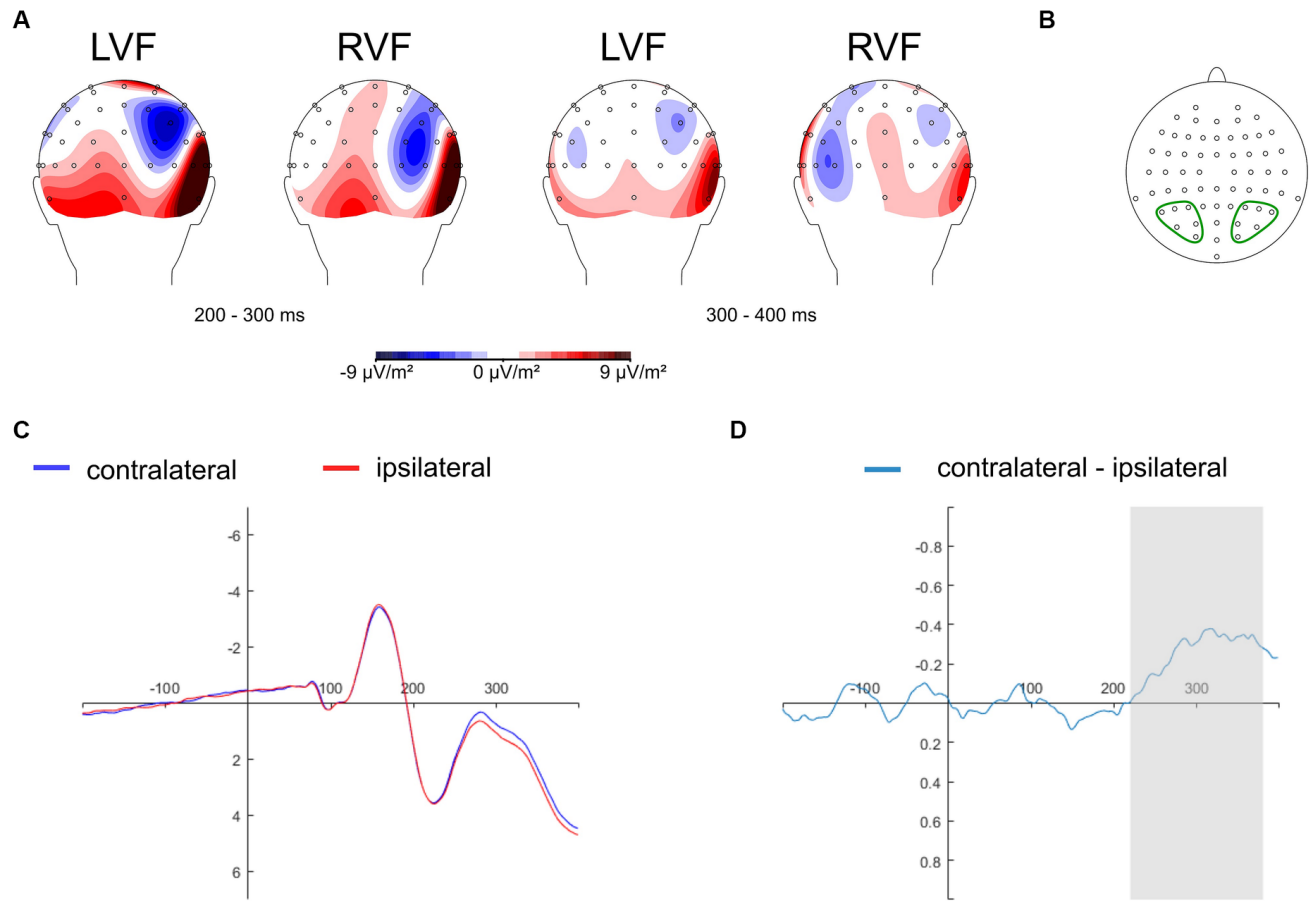
Topographical maps depicting the distribution of activity in the N2pc time window, derived by subtracting non-aligned trials from aligned trials (i.e., those containing cues), aggregated across all experimental conditions (self-face, close-other’s face, self-assigned shape, shape assigned to a close-other). There are two sets of maps illustrating amplitude distributions in two time intervals: 200–300 ms (left side) and 300–400 ms (right side), for cues presented in the left (LVF) and right (RVF) visual fields (Panel **A**). Electrode sites selected for analysis and pooled within the left and right parietal-occipital region: P3/P4, P5/P6, P7/P8, PO3/PO4, PO7/PO8, O1/O2 (panel **B**). The grand average ERPs are depicted for pooled electrodes positioned contralateral and ipsilateral to the cues, averaged across experimental conditions: self-face, close-other’s face, self-assigned shape, shape assigned to a close-other (Panel **C**). The contralateral-minus-ipsilateral difference wave (N2pc) is presented for all experimental conditions combined (Panel **D**). The gray shading designates the time interval (220–380 ms after the onset of cues) selected for the subsequent N2pc analysis.

The N2pc component is commonly studied at electrodes situated in the parietal-occipital region, known to yield maximal N2pc amplitudes, such as PO7/PO8 (Eimer and Kiss, 2008; Jolicœur et al., 2008; Kiss et al., 2008; Wójcik et al., 2018, 2019), and P7/P8 (Kappenman et al., 2014, 2015; Bola et al., 2021). Recent research has considered a range of electrode sites within a predefined parietal-occipital cluster of interest, including P1/P2, P3/P4, P5/P6, P7/P8, P9/P10, PO3/PO4, PO7/PO8, with data averaged across those sites (Papaioannou and Luck, 2020). In the present study, we adopted the collapsed localizer approach (Luck and Gaspelin, 2017) to select electrodes for ERP analysis in an unbiased manner. Thus, topographical activity distribution maps within the N2pc time window (200–400 ms) were aggregated across all experimental conditions: self-face, close-other’s face, self-assigned shape, and shape assigned to a close-other (Figure 3A). These maps were generated as follows: non-aligned trials were subtracted from trials featuring salient cues (related to self and close-other) presented in both the right and left visual fields. Subsequently, electrode sites were chosen for further analysis. They were situated within the regions of minimal activity (as the N2pc is a negative ERP component) in the parietal-occipital region of the hemisphere contralateral to the visual field of cue presentation. These sites included P3/P4, P5/P6, P7/P8, PO3/PO4, PO7/PO8, and O1/O2 (Figure 3B). The data were separately averaged for electrode sites located within the left and right parietal-occipital regions. This pooling of data is motivated by the inherent limitations of EEG’s spatial resolution and the strong correlation between neighboring electrodes. Furthermore, this strategy is often employed to streamline analysis and mitigate the potential for Type I errors (Papaioannou and Luck, 2020).

To isolate and visually represent the N2pc response to stimuli linked to the self and close-other, contralateral-minus-ipsilateral difference waveforms were calculated for each corresponding cluster of electrodes symmetrically situated in the left (P3, P5, P7, PO3, PO7, O1) and right (P4, P6, P8, PO4, PO8, O2) parietal-occipital region, for each experimental condition (self-face, close-other’s face, self-assigned shape, shape assigned to a close other). The ipsilateral and contralateral waveforms were determined as follows. The ipsilateral waveform was calculated as the average of signals from individual electrodes within the left parietal-occipital cluster for left-sided cues (self-face/shape, close-other’s face/shape), and corresponding electrodes within the right parietal-occipital cluster for right-sided cues. The contralateral waveform was calculated by averaging signals from individual electrodes in the left parietal-occipital cluster for right-sided cues, and the average of signals from corresponding electrodes in the right parietal-occipital cluster for left-sided cues. Subsequently, the difference waveforms were computed and averaged across all the selected cluster of electrode.

The collapsed localizer approach (Luck and Gaspelin, 2017) was also employed to pinpoint a suitable time window for measuring the N2pc. Thus, ipsilateral and contralateral waveforms were computed for pooled electrodes placed contralateral and ipsilateral to the cues and averaged across all experimental conditions: self-face, self-assigned shape, close-other’s face, and shape assigned to close-other’s (Figure 3C). Subsequently, the contralateral-minus-ipsilateral difference wave was calculated, and based on this difference wave (i.e., N2pc), the time window of 220–380 ms was selected (Figure 3D). Spanning 160 ms, this window covered the period of maximal changes in N2pc amplitude. This time window selection is in line with previous studies indicating that the N2pc component is typically evoked within the post-stimulus latency range of 200 to 400 ms (e.g., Kiss et al., 2008; Buodo et al., 2009; Holmes et al., 2009; Wójcik et al., 2019; Bola et al., 2021).

Subsequently, in order to evaluate the presence of the N2pc component, the mean amplitudes of ipsilateral and contralateral waveforms within the defined time window were directly compared through paired-samples t-tests. These tests were performed separately for each experimental condition (self-face, close-other’s face, shape assigned to self, shape assigned to close-other). Notably, significant differences between ipsilateral and contralateral waveforms were regarded as indicators of the N2pc component’s presence, while nonsignificant differences indicated its absence. For our analyses, non-aligned trials were excluded due to their lack of a laterally-located cue serving as a reference. In t-test outcomes, the mean difference between contralateral and ipsilateral waveform amplitudes was presented with 95% confidence intervals (CIs). Mean N2pc amplitudes (*M*) along with their corresponding standard deviations (*SD*) were reported as *M ± SD*. The traditional null-hypothesis significance-testing approach was complemented with Bayesian approach and Bayes factors (BFs) were computed. All statistical analyses were performed using the JASP software (Wagenmakers et al., 2018).

## 3. Results

### 3.1. Behavioral results

#### 3.1.1. Accuracy rates

High levels of accuracy were observed across all conditions of the dot-probe task. The accuracy percentages for congruent and incongruent trials were as follows: self-face (97.4 ± 3.1 and 97.4% ± 3.3%), self-assigned shape (98.1 ± 2.2 and 96.5% ± 4.4%), close-other’s face (97.9 ± 2.5 and 96.7% ± 3.8%), and shape assigned to close-other (97.1 ± 3.3 and 97.2% ± 3.3%). However, statistical analysis of accuracy values was not conducted due to the ceiling-level performance exhibited by the majority of participants.

#### 3.1.2. RTs

RTs for congruent and incongruent trials in all experimental conditions are provided in Table 1. Results of t-tests performed for each experimental condition indicated that differences between congruent and incongruent trials were non-significant (self-face: *t*(34) = −1.442, *p* = 0.164, 95% *CI* = [−9.436, 1.667]; close-other’s face: *t*(34) = −0.386, *p* = 0.702, 95% *CI* = [−7.825, 5.328]; self-assigned shape: *t*(34) = −0.537, *p* = 0.595, 95% *CI* = [−6.666, 3.880]; shape assigned to a close-other: *t*(34) = −1.158, *p* = 0.255, 95% *CI* = [−8.314, 2.277]). In turn, BFs were as follows: self-face – *BF_10_* = 0.455, close-other’s face – *BF*10 = 0.207, self-assigned shape – *BF_10_* = 0.194, and shape assigned to a close-other – *BF_10_* = 0.336. In general, all BFs indicated weak or moderate evidence in favor of H0.

**Table 1:** Reaction times (RTs) in each experimental condition.

Condition	Congruency	Md (ms)	SD
Self-face	congruent	482.604	86.766
	incongruent	486.489	80.031
Shape assigned to the self	congruent	456.150	72.217
	incongruent	457.543	72.137
Close-other’s face	congruent	479.953	68.170
	incongruent	481.201	72.310
Shape assigned to a close-other	congruent	455.024	66.032
	incongruent	458.043	61.718

### 3.2. N2pc results

The presence of N2pc (i.e., significantly lower amplitudes of the contralateral waveforms than of the ipsilateral waveforms) was evidenced for the self-face (1.102 ± 2.391 vs. 1.598 ± 2.511, *t*(30) = −4.342, *p* < 0.001, 95% *CI* = [−0.729, −0.263], *d* = −0.780), close-other’s face (1.276 ± 2.623 vs. 1.527 ± 2.638, *t*(30) = −3.732, *p* < 0.001, 95% *CI* = [−0.389, −0.114], *d* = −0.670), and self-assigned shape (2.555 ± 2.935 vs. 2.850 ± 2.866, *t*(30) = −2.195, *p* = 0.036, 95% *CI* = [−0.570, −0.021], *d* = −0.670). However, no N2pc was present for the shape assigned to a close-other (2.496 ± 2.497 vs. 2.491 ± 2.478, *t*(30) = 0.073, *p* = 0.942, 95% *CI* = [−0.154, 0.166], *d* = 0.013). In turn, BFs were as follows: self-face – *BF_10_* = 180.998, close-other’s face – *BF_10_* = 40.355, self-assigned shape – *BF_10_* = 1.537, and shape assigned to a close-other – BF_10_ = 0.192. While the two former BFs indicated extreme and very strong evidence in favor of H1, BFs in the case of a close-other’s face – weak evidence in favor of H1. In contrast, BF for the shape assigned to a close-other indicated weak evidence in favor of H0.

As the N2pc was present in the case of a self-assigned shape and absent in the case of a shape assigned to close-other we run an additional analysis: mean amplitudes of difference waves in the N2pc time-window were directly compared for this two conditions. It turned out that they significantly differed (*t*(30) = −0.226, *p* = 0.034, 95% *CI* = [−0.577, −0.025], *d* = −0.400, *BF_10_* = 1.625 – weak evidence in favor H1).

**Figure 4:**
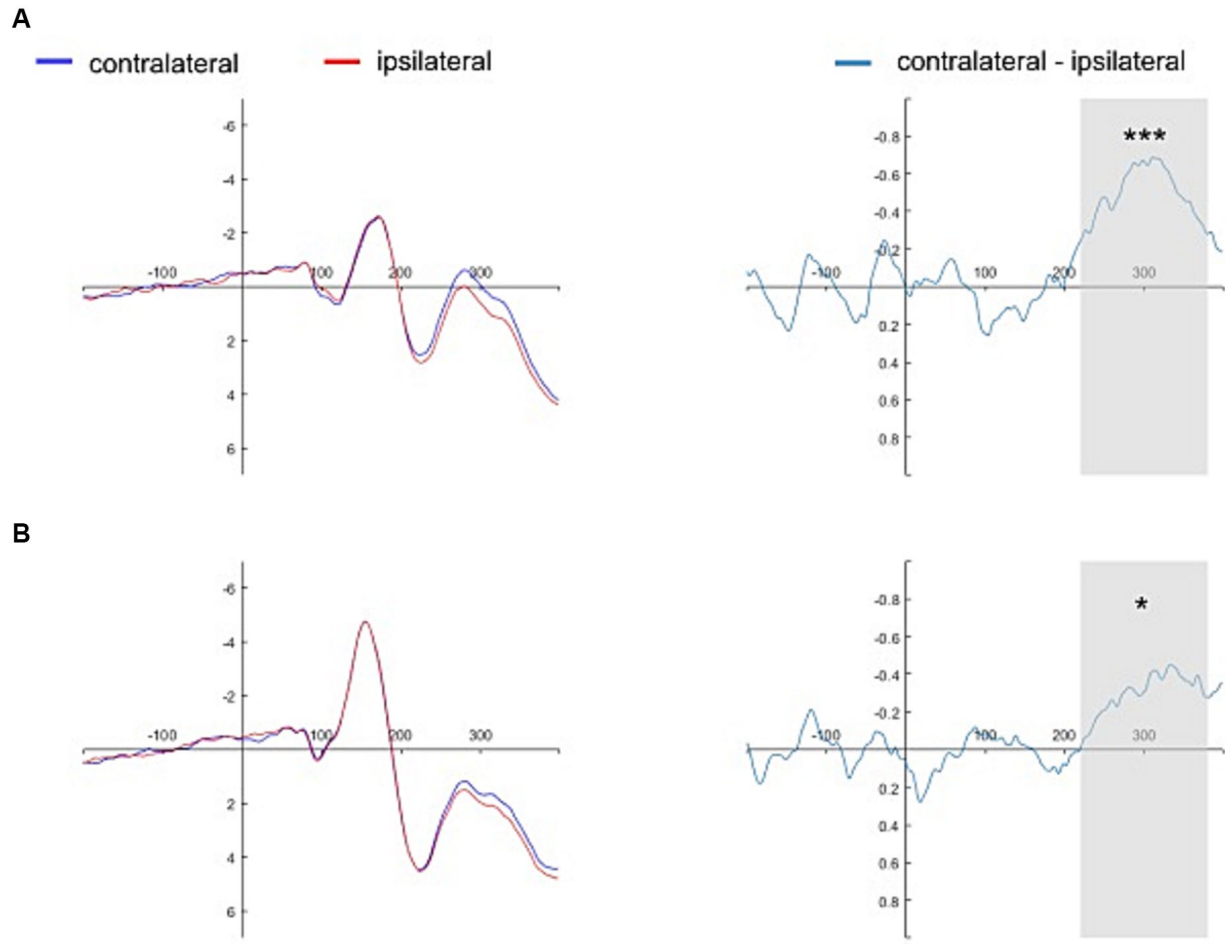
Grand average ERPs time-locked to the onset of self-referential cues. The grand average ERPs are displayed for pooled electrodes situated contralateral and ipsilateral to the self-face, along with the contralateral-minus-ipsilateral difference waveform, i.e., N2pc (Panel **A**). Similarly, the grand average ERPs for pooled electrodes placed contralateral and ipsilateral to the self-assigned shape are shown, accompanied by the contralateral-minus-ipsilateral difference waveform, i.e., N2pc (Panel **B**). The gray shading delineates the N2pc time window (220–380 ms following cue onset). Significant results are marked by asterisks: ***p < 0.001, *p < 0.05.

**Figure 5:**
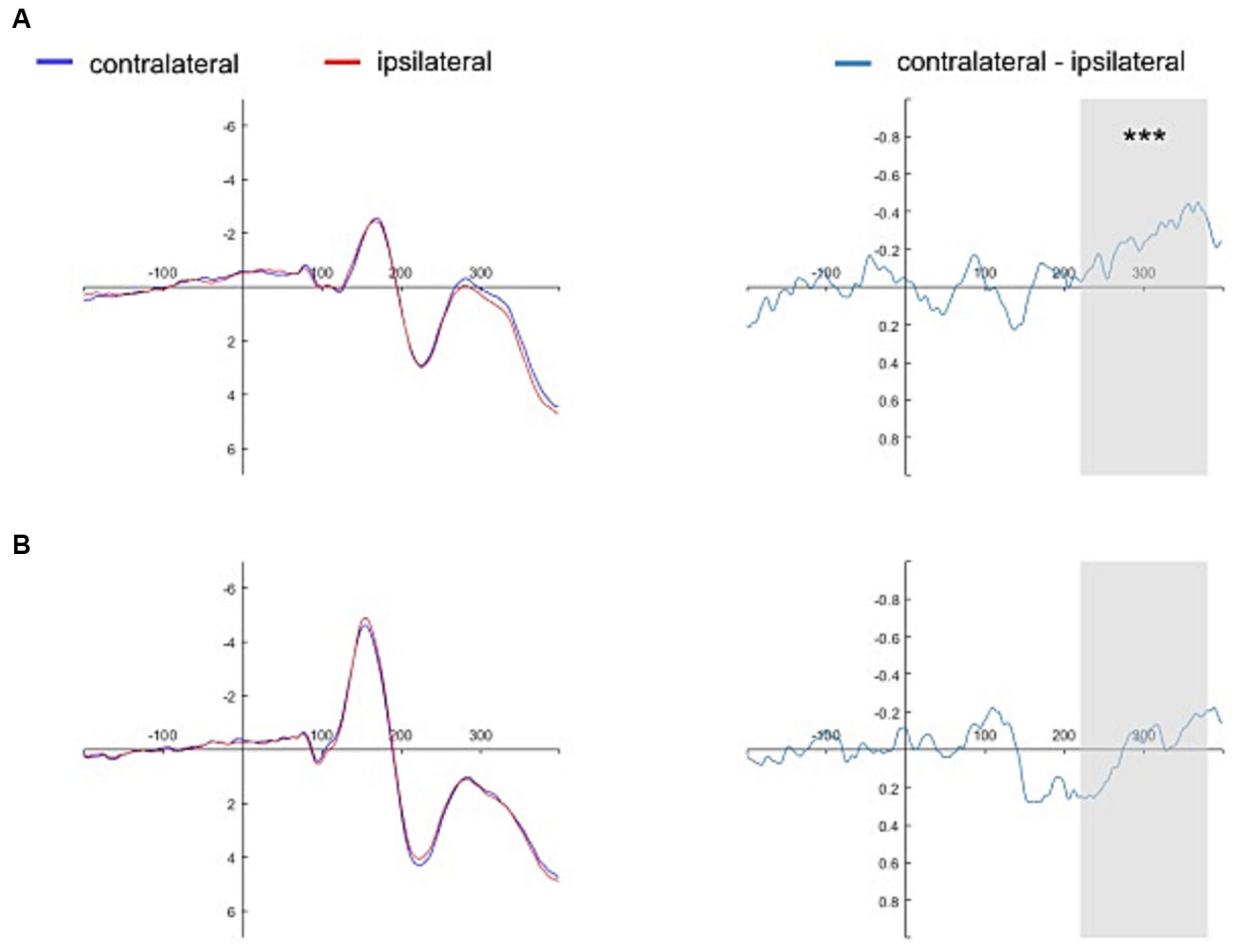
Grand average ERPs time-locked to the onset of close-other referential cues. The grand average ERPs are displayed for pooled electrodes situated contralateral and ipsilateral to the close-other’s face, along with the contralateral-minus-ipsilateral difference waveform, i.e., N2pc (Panel **A**). Similarly, the grand average ERPs for pooled electrodes placed contralateral and ipsilateral to the shape assigned to a close-other are shown, accompanied by the contralateral-minus-ipsilateral difference waveform, i.e., N2pc (Panel **B**). The gray shading delineates the N2pc time window (220–380 ms following cue onset). Significant result is marked by asterisks: ***p < 0.001.

Figures 4, 5 show the grand average contralateral and ipsilateral waveforms and the corresponding difference waveforms (i.e., N2pc) obtained across all experimental conditions. Specifically, Figure 4 illustrates results for the self-face and self-assigned shape conditions, while Figure 5 depicts outcomes for close-other’s face and shape assigned to a close-other conditions.

## 4. Discussion

In the present study, a dot-probe experimental paradigm was utilized to induce and measure attentional capture by task-irrelevant, to-be-ignored stimuli related to the self and the close-other. These stimuli came in two types: highly familiar and newly learned. Thus, the scope of the preferential processing of self-associated information was explored by directly addressing the questions of whether: (1) the processing of newly acquired self-referential information is facilitated at early processing stages, as is the case for the self-face; (2) both extremely familiar but not self-referential information (i.e., a close-other’s face) and new information assigned to a close-other also benefit from such prioritized attentional processing. The answers to these questions referring to attentional capture were based on evidence indicating the presence or absence of the N2pc ERP component, quantified as a significant difference between contralateral and ipsilateral (with respect to potentially salient cues, i.e., self/close-other’s face/shape) waveforms. Investigations aimed at answering the above questions may shed more light on the role of familiarity in the well-documented self-prioritization effect.

We have found ERP evidence indicating that both highly familiar (self-face) and newly acquired self-referential information (self-associated shape), as well as highly familiar but non-self-referential information (close-other’s face), automatically captured attention. This was revealed by the presence of the N2pc component. However, a shape associated with close-other did not elicit a similar effect, as evidenced by the absence of the N2pc. Moreover, direct comparison of amplitudes in the N2pc time window in two conditions involving shapes (shape assigned to the self vs. shape assigned to a close-other) revealed that they significantly differed. In general, our N2pc findings underscore the significance of extreme familiarity and self-reference factors in the automatic capture of attention and are comprehensively discussed below. Our N2pc results indicate that the self-face, a close-other’s face, and a self-assigned shape differed from a shape associated with a close-other in respect of their saliency. The former stimuli emerged as salient, in contrast to the latter which did not. This interpretation is grounded in N2pc findings from studies involving other salient stimuli, i.e., fearful faces (e.g., Eimer and Kiss, 2007).

Here, we have observed a dissociation between the behavioral and neural indicators of attention shifts, specifically the presence of the N2pc effects, while the RTs effects were absent. This effect corroborates findings from numerous preceding dot-probe studies involving the self-face (Wójcik et al., 2018, 2019; Bola et al., 2021) and other salient stimuli like emotional images (Kappenman et al., 2014, 2015). It is important to emphasize that the RTs for a manual response reflect the cumulative outcome of an entire sequence of processes occurring between stimulus presentation and response. Consequently, the RTs metric might not possess the necessary sensitivity to indicate transient and automatic attention shifts. Conversely, ERPs offer a continuous gauge of neuronal engagement, thus exposing concealed attention shifts that might not be evident in overt behavior. Supporting this rationale, prior research has demonstrated that the RTs index shows low internal reliability in the dot-probe task, and therefore its external validity is also likely limited whereas the N2pc’s reliability is moderate (Kappenman et al., 2014). In addition, the lack of RTs effects in our study (i.e., no differences between congruent and incongruent trials) may be related to relatively small group of tested participants. This supposition seems plausible taking into account that estimation of required sample size was based on electrophysiological results of our previous N2pc study (Bola et al., 2021). Nevertheless, it is worth noting that in regard to RTs all BFs provide weak or moderate evidence in favor of null hypothesis (Wagenmakers et al., 2018).

The N2pc results presented here for the self-face can be considered a form of conceptual replication of previously documented findings (Wójcik et al., 2018, 2019; Bola et al., 2021). In contrast, the presence of the N2pc component linked to the presentation of to-be-ignored self-assigned shapes represents a novel finding. The disparity between these two stimuli is noteworthy. An individual’s own face is not only an incredibly familiar but also a uniquely self-referential stimulus, likely constituting their most distinctive physical feature of the self (Tsakiris, 2008). The self-face is even regarded as a symbol of the self in its entirety (McNeill, 1998). In contrast, a self-assigned shape begins as an unfamiliar and irrelevant abstract stimulus, arbitrarily linked to one’s own identity. As a result, it does not hold a well-established role in the internal self-representation. Despite this contrast, both stimuli elicited a consistent N2pc component. This observation aligns with the perspective that self-reference heightens the social significance of stimuli by activating the self-concept. This activation, in turn, influences the cognitive processing of incoming information (Sui and Humphreys, 2015). It is worth stressing that automatic attention capture by a close-other’s face – evidenced by the presence of the N2pc component – has also been not previously reported.

In our earlier study (Żochowska et al., 2023), the processing of self-face, a close-other’s face, self-assigned shape, and shape assigned to a close-other was also investigated in comparison to the processing of unfamiliar faces and shapes. However, in that study all stimuli were in the focus of participants’ attention and participants judged whether presented stimuli were familiar or unknown. P3 results of that study showed preferential attentional allocation to the self-face (higher P3 amplitudes to the self-face than to close-other’s and unknown faces) and similar allocation of attentional resources to shapes assigned both to the self- and a close-other (similar P3 amplitudes). In contrast, in the present study faces and shapes were to-be-ignored stimuli (i.e., task-irrelevant distractors) and participants’ attention was directed toward a dot that followed presentation of face/shape pairs. Nevertheless, we found that that the self-face, a self-assigned shape, and a close-other’s face all captured attention automatically (as revealed by the presence of the N2pc). However, a shape assigned to a close-other did not attract attention (the N2pc was absent). In sum, while in the case of newly acquired information the previous study reported similar effects for shapes assigned to the self and a close-other, the current study showed dissimilar effects for those two types of stimuli.

One may speculate that this difference is related to different factors playing a role in bottom-up and top-down attention, reflected in the N2pc and P3 components, respectively. Results of our previous study (Żochowska et al., 2023) may suggest that top-down attention seems to be driven by the familiarity factor. However, an earlier ERP study with novel stimuli (unknown faces) associated with the self and others reported that self-associated faces led to higher P3 than friend- and stranger-associated faces, thus suggesting that the P3 effect is not (necessarily) driven by familiarity (Woźniak et al., 2018). Yet another ERP study (with perceptual shape-label matching task) also reported increased P3 component for self-assigned shape in comparison to shapes assigned to a friend and a stranger (Sui et al., 2023).The lack of consistency between findings of those P3 studies may result from procedural differences (e.g., different tasks, presence or absence of labels with established meaning). In turn, our N2pc results for the self-face and self-assigned shape may suggest that bottom-up attention is guided by the saliency of those stimuli (Sui and Humphreys, 2015). Specifically, it was proposed that self-reference enhances the salience of stimuli through the activation of the self-concept and this may modulate cognitive processes.

Furthermore, the N2pc findings concerning a self-assigned shape and a close-other’s face might be linked to the concept of the extended self. The self-concept extends beyond solely the physical or psychological self, encompassing a much broader scope. William James defined the self as the “sum of things that the person calls his or hers” (James, 1890, p. 291). In this context, the term ‘things’ implies all concepts, objects, names, social roles, semantic knowledge, and more, which are perceived as self-referential. In accordance with this perspective, social identity theory (Tajfel and Turner, 1986) and symbolic self-completion theory (Wicklund and Gollwitzer, 1981) propose that objects contribute to the symbolic definition of the self. In other words, individuals fuse objects with their own identity and integrate them into their internal self-image. Despite a substantial body of evidence indicating the prioritized processing of such objects, the underlying mechanism and its extent remain less understood (Ye and Gawronski, 2016). By adopting an associative approach, this relationship can be conceived as a mental link between the node representing the self and the node representing the object (Beggan, 1992; Gawronski and Payne, 2010), thus determining self-object linking. Greenwald et al. (2002) proposed that these mental associations form a larger associative network, composed of interconnected conceptual nodes centered around the self-node. These associative links facilitate the automatic spread of activation among nodes, meaning that the activation of a single node automatically triggers the activation of its interconnected nodes. Thus, the strength of these associations can be understood as the degree of ease with which activation spreads between nodes.

The extended self-concept envisions that not only certain objects but also certain other people are seen to be a part of the self (Belk, 1988, 2016). The concept of the extended self encompasses attributes related to group memberships, relationships, and social roles. It comprises those elements of the self-concept that distinguish between in-group and out-group members (Brewer and Gardner, 1996; Sedikides and Brewer, 2001; Sedikides et al., 2011). Achieving this extended sense of self is fundamental as a “need to belong,” inherent to human nature (Baumeister and Leary, 1995). The cumulative network model of the self proposes that the self is intrinsically relational across psychological, cultural, semantic, and social dimensions (Wallace, 2019). Hence, the self should be perceived as a socially constructed, embodied entity that engages with and extends into the social world and may be viewed as a network of relations. In addition, it has been suggested that the self extends to encompass external objects and individuals capable of eliciting emotions in agents (Heersmink, 2020).

Such an emotional attitude implies inclusion of those objects or people in the concept of extended self. It is worth noting that in the present study the close-other was freely chosen by each participant as a person who was for them the most important person at the time of experimentation. Thus, the close-other was not only a highly familiar person but also subjectively highly significant. Therefore, one may assume that images of a close-other’s face were as salient stimuli as images of one’s own face. In contrast to new information associated with a close-other, highly familiar information referring to a close-other may be viewed as a part of the extended self.

At this point, a question may arise of whether the N2pc results for a self-assigned shape may – at least partially – be explained by repeated presentations of that shape, a procedure that is typical for any ERP study. Such repeated presentations could potentially enhance the visual familiarity of the displayed stimuli. Nevertheless, if mere visual familiarity was sufficient to evoke N2pc, similar effects would be observed for any repeated shapes. However, this was not the case and the presence of N2pc ERP component (i.e., the evidence of early automatic attention capture) was clearly evident only for self-assigned shapes and not for shapes assigned to a close-other.

The N2pc component’s absence in shapes associated with a close-other is consistent with the findings from our previous dot-probe investigation concentrating on the impact of mere visual familiarity with faces (Bola et al., 2021). In that study, there was no indication of attention being captured by a face that became visually familiar due to repeated displays. This implies that visual familiarity does not hold the foremost role in driving attentional prioritization. Consistent with this notion, our current study demonstrates that newly acquired self-referential information was given a preference for attentional processing in the early stages, underscoring the significance of self-relevance.

One might ponder whether the disparate N2pc findings for shapes assigned to the self and those designated for a close-other could be linked to the varying efficacy in encoding novel information. Data encoded in relation to the self tends to be better retained than information encoded with other references, a phenomenon known as the Self-Reference Effect – SRE (Rogers et al., 1977; Symons and Johnson, 1997). The SRE has been observed across a range of stimuli, encompassing trait adjectives and nouns (Symons and Johnson, 1997) as well as visual objects (Hamami et al., 2011). Theoretical explanations posited for the SRE suggest the existence of established networks of knowledge and memories tied to the self. These networks are accessed during self-referential processing, facilitating more methodical and intricate processing than other information processing methods (Keenan et al., 1992; Symons and Johnson, 1997; Klein, 2012; Klein and Nelson, 2014). Consequently, self-referential encoding forms a more enriched, intricate memory trace that can be drawn upon for later recollection (Lawrence and Chai, 2021). While certain theories posit that forming new associations in memory is a relatively gradual process, necessitating repeated concept co-occurrences (Smith and DeCoster, 2000), some researchers contend that it is a relatively swift process grounded in minimal experiences (e.g., Gawronski and Bodenhausen, 2006, 2011). Thus, one may assume that a highly efficient self-referential encoding may – to some extent – contribute to the presence of the N2pc to self-assigned shapes.

Finally, findings of the present study may provide some arguments in an ongoing debate on the topic of factors critical for the emergence of prioritization of newly acquired self-referential information. There has been a suggestion that self-prioritization is inherently contingent on tasks or goals (Golubickis and Macrae, 2022). This idea was rooted in observations from studies demonstrating that self-relevance enhanced stimulus processing solely in tasks demanding overt self vs. other differentiation (e.g., Falbén et al., 2019; Caughey et al., 2021; Woźniak and Knoblich, 2022; Żochowska et al., 2023). For instance, it was demonstrated that self-prioritization became apparent when participants were tasked with reporting whom the stimulus represented, yet not when they were to report the spatial position (above or below a fixation cross) of a self-assigned shape (Caughey et al., 2021). Similarly, objects arbitrarily owned by the self were categorized more swiftly than those owned by a friend when participants were instructed to indicate either the owner or the identity of the items (Falbén et al., 2019). Conversely, self-relevance failed to enhance performance when participants judged the orientation of the stimuli. Along similar lines, the self-prioritization effect was observed solely when self-associations were pertinent to the task, and it was absent when self-associations were task-irrelevant (Woźniak and Knoblich, 2022). Nevertheless, in the current study we found automatic attention capture by a self- but not close-other assigned shape that was task-irrelevant and a to-be-ignored stimulus. This finding undermines the notion that self-prioritization of newly acquired self-referential information is task-dependent (Golubickis and Macrae, 2022).

Last but not least one may wonder whether results of this study may be viewed in the context of recent studies pointing to the role of valence in the emergence of self-prioritization. For instance, it has been reported that self-prioritization disappears when participants associate themselves with negatively connoted stimuli (Vicovaro et al., 2022; Lee et al., 2023). Specifically, when the self was paired with positive information (smiling faces, high reward) a clear evidence of a self-preference was found. In turn, pairing the self with less positive information (neutral faces, low reward) resulted in the lack of self-preference (Lee et al., 2023). Yet a more relevant study examined the impact of the valence on the self-prioritization effect by manipulating the valence of the visual shapes (either symmetrical or not), arbitrarily associated with the self and a stranger (Vicovaro et al., 2022). The self-prioritization effect occurred when the self was associated with symmetric shapes and the stranger with asymmetric shapes, whereas no evidence of a self-prioritization effect emerged for the opposite association. Taking into account that all but two shapes used in the present study were symmetrical and they randomly (on the group level) associated with the self, our results for the self-assigned shape corroborate those findings.

Future studies may examine automatic attention capture by other (than abstract shapes) novel information arbitrarily linked with the self and others. For instance, some previous studies (with different research questions and different experimental paradigms) investigated the processing of (initially unknown) faces associated with the self and close-other (Woźniak and Hohwy, 2020) or colors assigned to the self, friend, and stranger (e.g., Sun et al., 2016). However, none of them was focused on the N2pc component. It is of great interest whether dot-probe studies with such novel stimuli will confirm (or undermine) findings obtained for abstract shapes reported in the present study.

In sum, the findings of the current study seem to reveal the preferential attentional processing of information referring to the extended self. Humphreys and Sui (2015) introduced a framework, the Self Attention Network (SAN), designed to elucidate the prioritized handling of self-referential information. On the basis of our N2pc findings, we posit that the SAN might hold relevance in the context of the extended self as well. Specifically, the SAN is grounded in the concept that an individual’s self-representation remains in a continual state of activation, thereby swiftly elicited by the presence of stimuli associated with self-representation. Consequently, the prioritized processing of self-referential information could be attributed to the rapid initiation of bottom-up orienting processes derived from a consistently engaged self-schema. We propose that such processes may be activated when incoming stimuli refer to the extended self.

## 5. Conclusion

Results of this study evidence automatic attentional capture by highly familiar, long-established information referring to the self and to a close-other as well as by new abstract information that is arbitrarily linked to the self. Thus, one may conclude that: (1) prioritized early attentional processing was driven not only by the factor of familiarity but also by the self-reference of stimuli; (2) automatic attention capture was evidenced for stimuli referring to the extended self.

## Data availability statement

The raw data supporting the conclusions of this article will be made available by the authors, without undue reservation.

## Ethics statement

The studies involving humans were approved by Human Ethics Committee of the Institute of Applied Psychology at Jagiellonian University (Cracow, Poland). The studies were conducted in accordance with the local legislation and institutional requirements. The participants provided their written informed consent to participate in this study.

## Author contributions

AŻ: Conceptualization, Writing – original draft, Writing – review & editing, Data curation, Formal analysis, Investigation, Visualization. MW: Conceptualization, Formal analysis, Writing – review & editing. AN: Conceptualization, Writing – review & editing, Funding acquisition, Project administration, Resources, Supervision, Validation, Writing – original draft.
